# Supplementary material for: Mild Lactic Acid Stress Causes Strain-Dependent Reduction in SEC Protein Levels
Source: Microorganisms. 2021 May 8;9(5):1014. doi: 10.3390/microorganisms9051014 (PMC8151770; doi:10.3390/microorganisms9051014)
Supplement: Supplementary file 1 [file microorganisms-09-01014-s001.zip › microorganisms-1176190-supplementary.pdf]

Supplementary

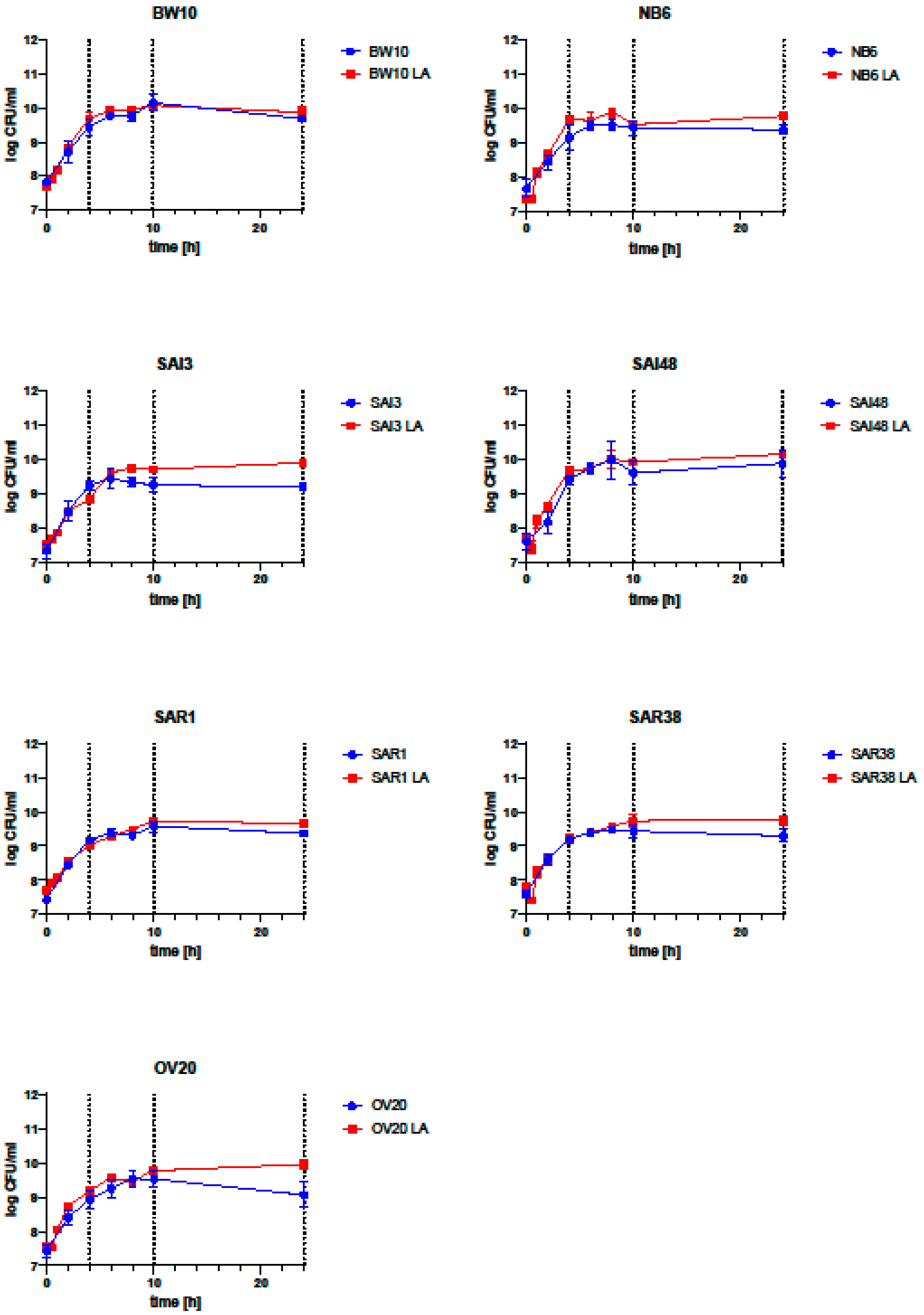

Figure S1 Growth curves under lactic acid stress and control conditions. Growth was compared for strains BW10, NB6, SAI3, SAI48, SAR1, SAR38, and OV20. Control conditions are shown in blue, mild lactic acid stress in red. Data points show mean values, error bars depict standard deviations.

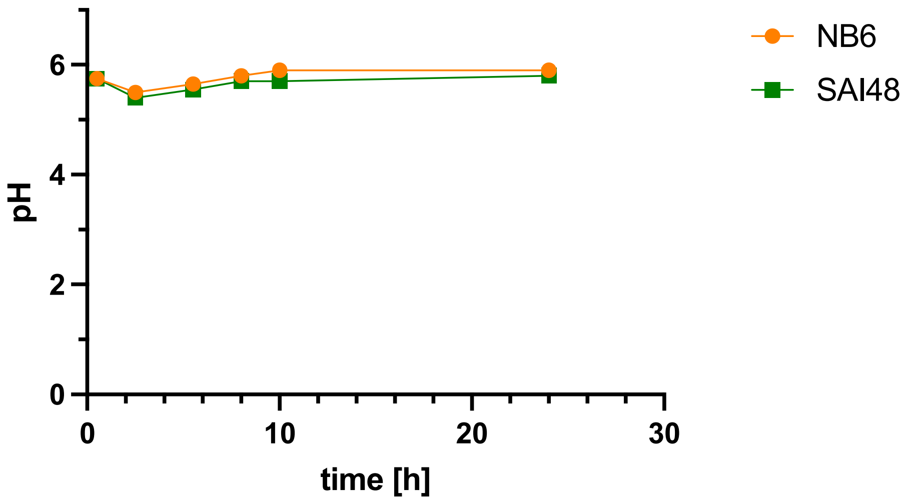

Figure S2 pH values for 2 representative strains NB6 (low toxin producer) and SAI48 (high toxin producer). NB6 is shown in orange circles, SAI48 in green squares. pH values consistently ranged between 5.4-5.9.
